# Flavour Profile of Traditional Dry Sausage Prepared with Partial Substitution of NaCl with KCl

**DOI:** 10.3390/foods12020388

**Published:** 2023-01-13

**Authors:** Li-Gang Qin, Xiang-Ao Li, Yu-Xiang Huang, Yong-Jie Li, Qian Chen

**Affiliations:** 1 College of Animal Science and Technology, Northeast Agricultural University, Harbin 150030, China; 2 College of Food Science, Northeast Agricultural University, Harbin 150030, China; 3 Branch of Animal Husbandry and Veterinary of Heilongjiang Academy of Agricultural Sciences, Qiqihar 161005, China

**Keywords:** sausage, sodium substitution, bacterial composition, sensory analysis

## Abstract

The effects of partial substitution of NaCl with 0%, 20%, 30% and 40% KCl on the physical characteristics, bacterial community and flavour profile of traditional dry sausage were investigated in this study. With the increase in KCl substitution ratio, the moisture content, astringency, bitterness and umami increased significantly, and the saltiness gradually decreased (*p* < 0.05). The high-throughput sequencing results showed that the dry sausages with KCl substitution had relatively high abundances of *Staphylococcus*. For volatile compounds, increasing the KCl substitution ratio reduced the formation of aldehydes, ketones and some alcohols, but promoted the formation of acids and esters (*p* < 0.05). Sensory evaluation and partial least square regression analysis showed that the dry sausages with 20% and 30% KCl were similar in overall physical and microbial properties, flavour profiles and sensory attributes, and the sausages with 40% KCl were characterized by taste defects. Overall, partial substitution of NaCl with 30% KCl could ensure the acceptable flavour and sensory attributes of dry sausages.

## 1. Introduction

Salt (NaCl) is an important curing ingredient in meat products. It improves the water retention and processing properties, inhibits harmful microbial growth and reproduction, confers saltiness as well as promotes characteristic flavour development [1,2]. However, most people consume an average of 9–12 g NaCl/day, which exceeds the reasonable adult consumption of <5 g/day recommended by the World Health Organization (WHO) [3,4]. High-sodium intake has become one of the three major dietary risk factors for cardiovascular disease, chronic kidney disease and osteoporosis, among other conditions [5]. Therefore, WHO member states have set a global target of a 30% reduction in salt intake by 2025 [6].

Meat products are one of the main sources of sodium, accounting for proximately 20–30%) [7,8]. Due to the specific role of NaCl in flavour perception (e.g., promoting saltiness and volatiles release, restraining bitterness) and quality improvement (e.g., improving texture and inhibiting undesirable microbial growth), approximately 2–3% NaCl is added during preparation, which rises to 3–5% NaCl due to the evaporation of water during long-term fermentation [9,10]. Thus, dry sausages need salt reduction strategies to meet the health needs of consumers.

One of the common salt reduction strategies is to use NaCl substitutes (e.g., magnesium, potassium and calcium salts), especially potassium chloride (KCl) [11], which has similar characteristics and equivalent antimicrobial properties to NaCl [10,12]. Furthermore, appropriately increasing potassium intake can reduce the risk of cardiovascular disease [13]. However, it is impossible to substitute NaCl with KCl completely because of the bitterness, astringency and metallic taste associated with high KCl addition (≥40%) [14,15]. Other than the taste attributes, the partial substitution of NaCl with KCl can affect the final volatile profiles for various reasons. Sodium and potassium have different inhibitory effects on protease activities and the oxidation of lipids and proteins [16,17,18], which greatly influences the development of volatile compounds; furthermore, the partial substitution of NaCl with KCl may change the composition and abundance of volatile compound-producing microorganisms (e.g., *Staphylococcus*) [19,20]. Therefore, in this study, we explored the maximum substitution of NaCl with KCl to guarantee quality and acceptability of dry sausages.

In summary, the comprehensive flavour profiles of dry sausages with KCl substitution should be paid more attention. In recent years, gas chromatography–mass spectrometry (GC–MS), combined with electronic tongue (E-tongue) and electronic nose (E-nose), has become an important method to obtain the overall flavour characteristics of meat products, such as Harbin red sausages [21], cooked chicken drumsticks [22] and smoked bacon [23]. The combination of these three techniques can provide complementary and comprehensive information to flavour analysis. Therefore, the present study evaluated the influence of partial substitution of NaCl with 0%, 20%, 30% and 40% KCl on the taste and odour properties of dry sausages using E-tongue, E-nose and GC–MS. The partial least squares regression (PLSR) model was employed to further explore the relationship among physical and microbial properties, key volatile compounds and sensory characteristics.

## 2. Materials and Methods

### 2.1. Materials

Fresh pork back fat and lean pork were purchased from Dazhuangyuan industrial Co. (Harbin, China) and were transported to a meat science laboratory at 4 °C. Spices, glucose, NaCl and KCl were food grade and purchased from the local fresh market. All chemicals were analytical grade and purchased from Solabio Corporation (Beijing, China).

### 2.2. Preparation of Dry Sausages

In accordance with the procedures described by Hu et al. [24], with some modifications, traditional dry sausage was manufactured from 2700 g of lean pork and 300 g of pork back fat minced through a 1.5-cm plate. The following additives were added to the minced meat in all treatments: 75.0 g of salt, 0.27 g of sodium nitrite, 30.0 g of wine, 30.0 g of glucose and 15.0 g of ginger powder, 9.0 g of monosodium glutamate, 150 g of water and 24.0 g of mixed spices (Shiyitang, Heilongjiang, China). The mixed spices consisted mainly of specific proportions of Pericarpium zanthoxyli, cassia bark, angelica, clove, fennel, round cardamom, Amomum villosum, pepper and aniseed. Four treatments of dry sausage were investigated, including the control treatment (0%: 2.50% NaCl), and three substitution treatments with different KCl substitution ratios (20%: 2.00% NaCl + 0.50% KCl; 30%: 1.75% NaCl and 0.75% KCl; 40%: 1.50% NaCl and 1.00% KCl).

After the minced meat and additives were fully mixed, the mixture was poured into porcine casings, with the length and diameter of each sausage approximately 15 and 2.5 cm, respectively. The sausages were hung in a controlled environment at 25 ± 2 °C and 30–50% relative humidity for 24 h, then transferred to an environmentally-controlled incubator at 25 ± 2 °C and 65–70% relative humidity for 11 days. The sausages were collected at days 0, 3, 6, 9 and 12 to analyse the physical characteristics and bacterial counts, at day 0 and 12 for analysis of the flavour profiles and bacterial community composition, and at day 12 for sensory evaluation.

### 2.3. Determination of Moisture Content, Water Activity and pH

The moisture content was determined using AOAC procedures [25]. The water activity (*a*_w_) was recorded via an Aqualab water activity meter (Decagon Devices, Pullman, WA, USA). For analysis of the pH, 10.0 g of sausage was homogenised with 90.0 mL of deionised water. After resting for 30 min at room temperature, the filtrate was collected and analysed using a pH meter (Mettler Toledo, Zurich, Switzerland).

### 2.4. Determination of Bacterial Counts

According to our previous study [26], the serial decimal dilutions of sausage sample homogenates were prepared. The counts of total aerobic bacteria and lactic acid bacteria (LAB) were determined on Plate Count Agar (at 37 °C for 48 h) and de Man Rogosa Sharpe Agar (at 37 °C for 72 h), respectively.

### 2.5. Bacterial Community Analysis

#### 2.5.1. Bacterial DNA Extraction and Sequencing

The bacterial DNA from dry sausage was extracted through the cetyltrimethyl ammonium bromide (CTAB) method [27]. The purity and concentration of the extracted DNA were detected by 1% agarose gels, then the DNA concentration was diluted to 1 ng/μL using sterile water. The V1-V9 regions of 16S rRNA genes were amplified using the specific forward primer (5′-AGAGTTTGATCCTGGCTCAG-3′) and reverse primer (5′-GNTACCTTGTTACGACTT-3′). The PCR condition was based on the procedures described of Wen et al. [26]. The PCR products were detected and purified by 2% agarose gel and QIAquick@ Gel Extraction Kit (QIAGEN, Hilden, Germany), respectively. The sequencing library was generated with SMRTbell TM Template Prep Kit (Pacific Biosciences, Menlo Park, CA, USA). Then, the library quality was assessed on Qubit @ 2.0 Fluorometer (Thermo Scientific, MA, USA). Finally, the library was sequenced on a Pacific Biosciences (PacBio, Menlo Park, CA, USA) sequence platform.

#### 2.5.2. Bioinformatic Analysis

To obtain clean reads, the raw reads were filtered by QIIME software (Version 1.9.1), and the chimera sequences were removed, based on the UCHIME algorithm. The sequence was clustered into operational taxonomic units (OTUs) with ≥97% similarity using Uparse software (Uparse v7.0.1001, http://drive5.com/uparse/, accessed on 4 February 2022), and representative sequences for each OTU were screened. The annotated taxonomic information of representative sequences was carried out based on the Mothur algorithm and SSUrRNA Database of Silva Database (http://www.arb-silva.de/, accessed on 20 March 2022).

### 2.6. Electronic Tongue Analysis

E-tongue analysis of the dry sausages was conducted, based on the method of Zhang et al. [22], with some modifications. Briefly, a mixture of 30.0 g of minced sausage and 150.0 mL of deionised water was incubated in a water bath at 40 °C for 30 min, then stirred in a blender (IKA T18 Basic, IKA-Werke GmbH & Co., Staufen, Germany) at low speed for 1 min. After centrifugation (5000× *g* at 4 °C for 10 min), the supernatant was collected and analysed by the E-tongue system (Intelligent Sensory Technology/Insent Company, Atsugi-shi, Japan) equipped with five taste sensors: AAE (umami), AE1 (astringency), CT0 (saltiness), CA0 (sourness), C00 (bitterness), and two reference electrodes.

### 2.7. Electronic Nose Analysis

The E-nose analysis of dry sausages used the portable E-nose system PEN3 (Airsense Analytics GmbH, Schwerin, Germany). The PEN3 system was equipped with 10 metal oxide semiconductors, which responded to the corresponding sensitive volatile substances (including W1C, W5C, W1S, W3C, W2S, W3S, W5S, W6S, W1Wand W2W). The information on ten electronic nose sensors were described with reference to Zhang et al. [22]. A total of 3.00 g of minced sausage was placed in a 20-mL headspace vial (CNW Technologies, Duesseldorf, Germany) and left to equilibrate at room temperature for 30 min. The volatile gas was pumped through the sensor array with a flow rate of 200 mL/min, and the measurement phase lasted 60 s.

### 2.8. Volatile Compound Analysis

The volatiles in dry sausages were extracted and analysed using a headspace solid-phase micro-extraction (HS-SPME) device (Supelco, Bellefonte, PA, USA) and a Shimadzu QP2020 GC-MS system (Kyoto, Japan) with an InertCap WaX (60 m × 0.25 mm × 0.25 μm) capillary column, respectively, as described by Wen et al. [18]. The volatiles were identified by comparison with the NIST 17 experimental mass spectra library and with the linear retention indices (LRI) of a series of standard alkanes (C6-C20). Identification was based on a similarity > 90%. Volatile compounds were semi-quantified by the internal standard method (expressed as μg/kg).

### 2.9. Sensory Evaluation

The sensory evaluation was performed following the method of Chen et al. [28] with some modifications. Twenty (10 male and 10 female) food professionals were selected and trained for sensory evaluation across three sessions. Training sessions were conducted on the operation of sensory evaluation and quality standards of different attributes before sensory evaluation. The 12-day fermented dry sausages were cooked (100 °C, 20 min), cut into 5-mm-thick slices and placed in a plastic plate randomly coded with 4 digits. The dry sausages were evaluated by a 7-point system, ranging from 1 (low intensity) to 7 (high intensity). Sensory attributes included aroma, hardness and taste (metallic taste, umami taste, salty taste, bitter taste and astringent taste).

### 2.10. Statistical Analysis

Three independent batches of dry sausages (replicates) were prepared, and all indices of each batch were measured in triplicate (triplicate observations). The results were expressed as mean ± standard error (SE). Data were analysed using the general linear model procedure of the Statistix 8.1 software package (Analytical Software, St Paul, MN, USA). Analysis of variance (ANOVA) with Tukey’s multiple comparison was used to assess the significance of the treatment effects (*p* < 0.05). Variation in physical properties and bacterial counts were described using a mixed model, with the different treatments (0%, 20%, 30% and 40% KCl) and fermentation times (0, 3, 6, 9 and 12 days) as fixed terms, and each replicate as a random term. For E-nose, E-tongue, bacterial community and volatile compound analysis, fixed terms for a mixed model included different treatments (0%, 20%, 30% and 40% KCl) and fermentation times (0 and 12 days), and a random term was each replicate. For sensory evaluation, a fixed term for a mixed model included different treatments (0%, 20%, 30% and 40% KCl), and random terms included sausage and sensory panel (session number, tasting order and panelist number). Principal component analysis (PCA) and PLSR model was performed using Origin 2019 (OriginLab Corporation, Northampton, MA, USA) and Unscrambler X (version 10.4, CAMO ASA, Oslo, Norway), respectively. Partial least squares discriminant analysis (PLS-DA) and variable importance projection (VIP) value was analysed with a free online platform (https://www.bioincloud.tech, accessed on 25 March 2022).

## 3. Results and Discussion

### 3.1. Moisture Content, Water Activity, pH and Bacterial Count Analysis

As shown in Table 1, the moisture content of dry sausages decreased significantly (*p* < 0.05) due to water loss during fermentation. After a 12-day fermentation, the moisture content of the 0%, 20%, 30% and 40% KCl substitution treatments decreased from approximately 67.22% to 18.02%, 18.67%, 18.84% and 19.95%, respectively (*p* < 0.05). An upward trend of moisture content occurred as the KCl substitution ratio increased, especially at day 3 and day 12 (*p* < 0.05), which was also found in the salted pork with KCl substituted for NaCl [29]. This phenomenon was probably due to the rapid penetration of the mixture of KCl and NaCl into the meat product, preventing the exit of water [30]. The delay in water evaporation was also observed in the dry sausages with KCl substitution combined with flavour enhancer [28]. The *a*_w_ of each treatment decreased rapidly (*p* < 0.05) from day 0 to day 9 and then tended to remain stable. The final *a*_w_ was 0.65–0.67, at which most microorganisms stop growing [31]. There were no significant differences among the KCl substitution treatments (except for the sausages on day 12) (*p* > 0.05).

The initial pH value of dry sausages was approximately 6.17, then decreased rapidly from day 0 to day 3 (*p* < 0.05). The cause might have been the growth and reproduction of LAB in the early stage of fermentation, and LAB metabolise carbohydrates and produce large quantities of organic acids under suitable environmental conditions [18]. From day 3, the pH gradually stabilised and then slightly increased, perhaps because of the accumulation of non-protein nitrogen and basic products of amino acid metabolism [32]. Overall, KCl substitution had no significant effect on the pH value of dry sausages (except for the sausages at day 9) (*p* > 0.05) [14,33].

The counts of LAB and total aerobic bacteria increased rapidly from day 0 to day 3 (*p* < 0.05) due to abundant nutrients and the high moisture content, reaching a maximum value at day 6 of >7 log CFU/g. From day 6, the counts of LAB and total aerobic bacteria began to decrease gradually as a result of the unsuitable fermentation environment [24]. Generally, there was no obvious change trend of bacterial counts during fermentation and significant differences (*p* > 0.05) among the final products.

### 3.2. Community Composition Analysis

**Figure 1:**
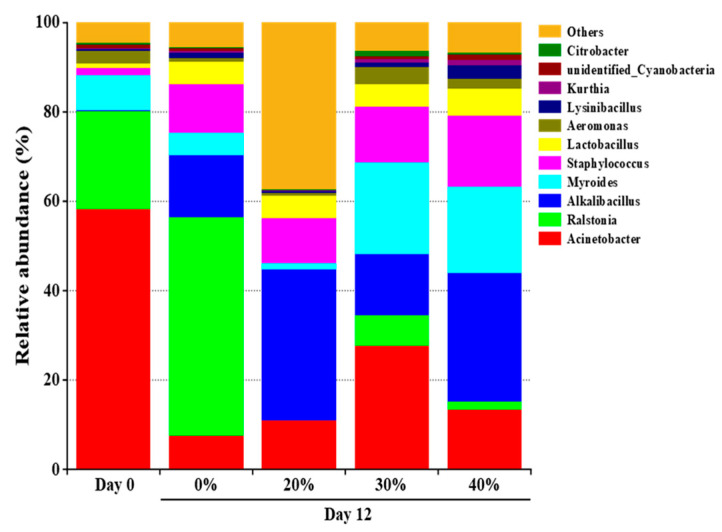
The relative abundance of dominant bacteria at the genus of traditional dry sausage with different KCl substitution ratios at day 0 and day 12. 0%: control dry sausage (2.50% NaCl); 20%: dry sausage with 20% KCl substituted for NaCl (2.00% NaCl + 0.50% KCl); 30%: dry sausage with 30% KCl substitution (1.75% NaCl + 0.75% KCl); 40%: dry sausage with 40% KCl substitution (1.50% NaCl + 1.00% KCl).

A total of 71 bacterial genera were found in the dry sausages, of which 11 genera, having a relative abundance over 1%, were dominant bacteria (Figure 1). The most abundant bacteria in the initial dry sausages included *Acinetobacter*, *Ralstonia* and *Myroides*, accounting for 58.16%, 22.00% and 7.97%, respectively. These genera were probably derived from the raw meat, spices, processing equipment and environment [27]. *Acinetobacter*, which exists widely in the environment and lacks important biochemical activities [34], was reported as the predominant genus in fresh Harbin red sausage [35] and the dorsal muscles of mandarin fish [36]. After 12 days of fermentation, the bacterial profile changed significantly. Obviously, the relative abundance of *Acinetobacter* decreased sharply, while the relative abundance of *Alkalibacillus*, *Staphylococcus* and *Lactobacillus* increased rapidly in all treatments. This was attributed to the tolerance of these microorganisms to high salt concentration, low pH and *a*_w_. Moreover, LAB can produce organic acids and bacteriocins during fermentation, which also inhibit the growth of other microorganisms [37]. The higher relative abundance of *Staphylococcus* observed in the KCl substitution treatments is noteworthy. Similar results were found in Harbin dry sausage, which might be due to the fact that K^+^ interferes less with intracellular metabolic activities and KCl substitution promotes the growth of salt tolerant bacteria (e.g., *Staphylococcus*) [19,20]. Previous studies showed that *Staphylococcus* was usually the dominant genus of fermented meat products [35,36] and responsible for the generation of flavour compounds, such as polypeptides, free amino acids, free fatty acids and esters [31,38].

### 3.3. Electronic Tongue Analysis

**Figure 2:**
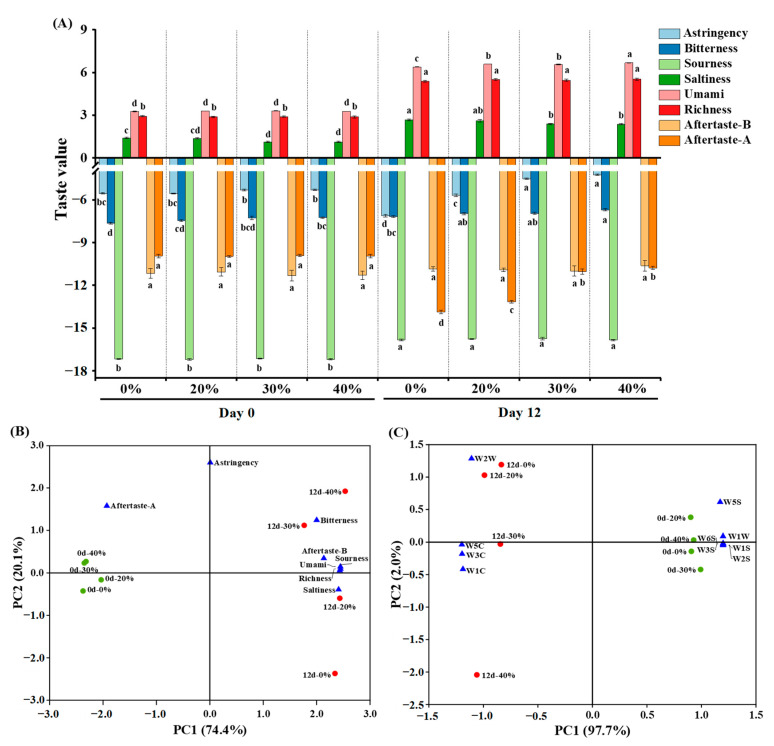
Taste assessment (**A**) and principal component analysis (**B**) based on the electronic tongue sensor responses, and principal component analysis (**C**) based on the electronic nose sensor responses of traditional dry sausage with different KCl substitution ratios at day 0 and day 12. ^a–d^ Means within the same taste attribute differ significantly among the different treatments (*p* < 0.05). 0%: control dry sausage (2.50% NaCl); 20%: dry sausage with 20% KCl substituted for NaCl (2.00% NaCl + 0.50% KCl); 30%: dry sausage with 30% KCl substitution (1.75% NaCl + 0.75% KCl); 40%: dry sausage with 40% KCl substitution (1.50% NaCl + 1.00% KCl).

As depicted in Figure 2A, at day 0, partial substitution of NaCl with KCl only had significant effects on the bitterness and saltiness (*p* < 0.05), with higher bitterness and lower saltiness observed in the 40% KCl treatment, compared to the other treatments, especially the control treatment. After a 12-day fermentation, the values of bitterness, sourness, saltiness, umami and richness increased (*p* < 0.05), while the aftertaste-astringency (aftertaste-A) decreased (*p* < 0.05). These changes in taste may be attributed to the decrease in moisture content, decomposition and oxidation of proteins and lipids, and the microbial activity of the dry sausages during fermentation, which led to the increased concentration and formation of taste substances (e.g., free amino acids, peptides) [39]. With the increase in the KCl substitution ratio, astringency, bitterness and umami gradually increased (*p* < 0.05), and saltiness gradually decreased (*p* < 0.05) at day 12. The changes in astringency, bitterness and saltiness were likely caused by the stronger bitterness, astringency and weaker saltiness of KCl than NaCl [40]. In addition, NaCl can also partially mask bitterness [41]. KCl may promote the production of umami substances (e.g., free amino acids, peptides) during fermentation, by affecting enzyme activity and microbial metabolism [17,42], which could explain the observed increase in umami.

PCA is a multivariate statistical tool for reducing the dimensionality of multivariant data through linear combinations. It can explain the regularity and differences between samples on the premise of retaining maximum variance of the original data [43,44]. As shown in Figure 2B, the total variance contributed by PC1 and PC2 was 94.5% (74.4% and 20.1%, respectively). All sausages at day 0 and day 12 were distributed in the negative and positive axes of PC1, respectively. The sausages at 0 day were clustered, due to a similar overall taste profile that was only related to the aftertaste-A. The overall taste profiles of the sausages at day 12 showed a dispersed distribution along the PC2 axis due to differences in terms of bitterness, aftertaste-bitterness (aftertaste-B), sourness, umami, richness and saltiness. The treatments with different KCl substitution ratios were far from each other, indicating that KCl had influenced their overall taste profiles.

### 3.4. Electronic Nose Analysis

As shown in Table 2, the response values of the W1S, W2S, W3S, W1W, W5S and W6S sensors at day 0 were higher than those at day 12 (*p* < 0.05), especially for W1S, W2S and W6S sensors. The relatively high response value of W2S sensor was related to the addition of wine during sausage preparation, and the response values of the 10 sensors of the E-nose showed no significant differences (*p* > 0.05) among treatments at day 0.

After a 12-day fermentation, W1C, W3C, W5C and W2W sensors had stronger responses, which indicated that the dry sausages had more aroma compounds (such as aromatic components and organic sulfides) than the sausages at day 0. This was consistent with the results of the volatile compound analysis (Section 3.5). Furthermore, the response values of W1C, W3C, W5C and W1W sensors were significantly different (*p* < 0.05) among treatments; W1C, W3C and W5C sensors showed an increasing trend of response values with the increase in the KCl substitution ratio, which was opposite to the trend of the W1W sensor, perhaps because of the differences in moisture content and metabolites among treatments.

As depicted in Figure 2C, the total variance contributed by PC1 and PC2 was 99.7% (97.7% and 2.0%, respectively). The dry sausages at day 0 and day 12 were distributed in the positive and negative axis of PC1, respectively, suggesting that their odours differed significantly. All dry sausages at day 0 were clustered in one group, and their odours were related to W1S, W2S, W3S, W5S, W6S and W1W sensors. By contrast, the dry sausages at day 12 were distributed from top to bottom on the PC2 axis, and their odours were related to W1C, W3C, W5C and W2W sensors. However, PCA, based on the E-nose response data, could not distinguish the differences of overall odour profiles in the sausages with different KCl substitution ratios at day 12 well since PC2 showed a little variance.

### 3.5. Volatile Compound Analysis

As shown in Table 3, a total of 54 volatile compounds were identified in dry sausages. The volatile compounds were mainly composed of aldehydes (3), ketones (5), alcohols (10), acids (5), esters (10), alkanes (3), alkenes (14) and others (4). Among the aldehydes detected, hexanal and nonaldehyde produced by polyunsaturated fatty acid oxidation [45,46] present the herbaceous, grass and pungent odours and are important contributors to the typical flavour of dry-cured meat products [47]. In this study, aldehydes presented lower contents in the KCl substitution treatments than that in the control treatment (*p* < 0.05). Hexanal is regarded as the indicator of lipid oxidation [48], and its contents in the sausages substituted with KCl were higher than in the control sausage, which may be explained by the oxidation ability of NaCl being promoted in the presence of KCl [29]. Nevertheless, the extent of oxidation seems to be related to the proportion of KCl substitution, as the highest hexanal content was exhibited in the sausages with 20% KCl substitution (*p* < 0.05).

A total of 5 ketones was determined in dry sausages. Among them, methyl ketones (2-butanone, 2-nonanone and 3-hydroxy-2-butanone) are representatives of the characteristic fermented flavour compounds [49]. The ketones 2-Butanone and 2-nonanone are generated by the lipid β-oxidation pathway in microorganisms, such as molds and *Staphylococcus* [49,50]. The ketone 3-Hydroxy-2-butanone is produced from LAB using citrate and pyruvate as substrates [51]. Partial KCl substitution increased the concentration of methyl ketones compared to the control treatment, which could be attributed to reduced inhibitory effects on microbial growth by partial replacement of NaCl with KCl. An increase in the abundance of ketones was also reported by Luo et al. in dry-cured lamb ham with 25% KCl substitution [46]. In addition, with the increase in KCl substitution ratio, the concentration of methyl ketones decreased significantly (*p* < 0.05). 

Alcohols are mainly derived from raw material (e.g., wine) and biosynthesis, including carbohydrate fermentation, methyl ketone reduction, amino acid metabolism and lipid oxidation [52]. Nevertheless, these compounds usually have relatively high threshold values. In this study, the secondary alcohol 1-octen-3-ol (mushroom odour) was found in all the sausages, which is an important aroma contributor with a low threshold value, derived from linoleic acid oxidation [53]. In addition, some alcohols (such as 2,3-butanediol, 2-ethyl hexanol and 2-heptanol) were present in the lower concentrations in the sausages with higher KCl substitution ratios.

Acids were detected only during the fermentation of the dry sausages. Among the five acids detected, acetic acid, derived from carbohydrate metabolism, and having a low threshold value, is related to the characteristic ripened aroma of dry sausages [54]. In general, the concentrations of acids (except for octanoic acid) increased dramatically with the increasing substitution of NaCl with KCl, especially in the 40% KCl treatment at 12 days. A similar result was also reported by Wu et al. [50] in dry-cured bacon. They found that the change could be related to higher microbial activities because of the decreasing Na^+^ content.

Esters are desirable compounds in dry sausages and have very low threshold values. Not only do they provide flower and fruit aromas to the fermented products, but they also mask rancid smells [55,56]. Esters are usually produced by the non-enzymatically catalysed esterification of alcohols and acids, as well as the enzymatic catalysis of esterification reactions under the action of bacteria (such as *Staphylococcus*) and fungi (yeasts and molds) during fermentation [57,58,59,60]. A total of 6 and 10 esters were detected at day 0 and day 12, respectively. Ethyl esters were the most abundant esters, followed by methyl esters. Meanwhile, γ-butyrolactone, a product of the dehydration of γ-hydroxylic acid [60], was the only lactone identified. In the present study, KCl substitution seems to promote the formation of esters, which may be due to the higher abundance of *Staphylococcus* and *Acinetobacter*, well known for their capacities in ester formation [55,61], in KCl substitution treatments. 

Alkanes are mainly produced by the molecular re-arrangement of peroxides during lipid autoxidation [49]. Due to high threshold values, alkanes have little effect on flavour perception [36], but alkanes, as the precursor of carbonyl compounds, have a potential flavour-promoting effect. Fourteen alkenes and four other compounds were identified and provided fresh and sweet smells in dry sausages [62]. Although some came from the meat as a result of their presence in animal feedstuffs [53], most, as well as small amounts of aldehydes (e.g., cinnamaldehyde), ketones (e.g., fenchone) and alcohols (e.g., linalool), originated from spices, such as pepper, fennel and clove. The concentration of these compounds increased greatly due to dehydration during fermentation.

### 3.6. Partial Least Squares Discriminant Analysis

**Figure 3:**
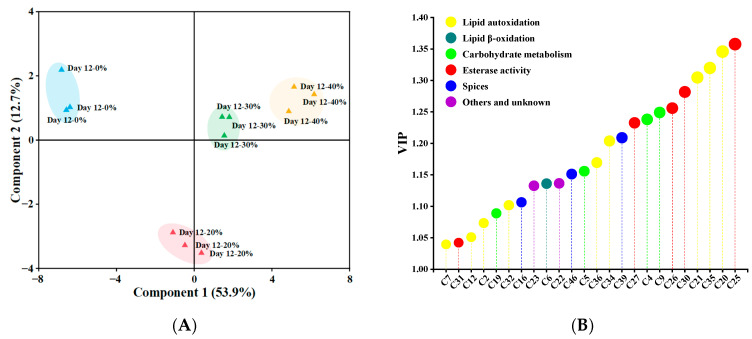
The partial least squares discriminant analysis score plots (**A**) and the variable importance in the projection (VIP) values (**B**) of significantly different (*p* < 0.05) volatile compounds in traditional dry sausages with different KCl substitution ratios at day 12. The size of circle represents VIP value, the larger VIP value, the larger the circle. 0%: control dry sausage (2.50% NaCl); 20%: dry sausage with 20% KCl substituted for NaCl (2.00% NaCl + 0.50% KCl); 30%: dry sausage with 30% KCl substitution (1.75% NaCl + 0.75% KCl); 40%: dry sausage with 40% KCl substitution (1.50% NaCl + 1.00% KCl).

The PLS–DA classification pattern, based on 39 significant differential (*p* < 0.05) volatile compounds, is presented in Figure 3A, distinguishing samples after 12 days of fermentation [63]. Component 1 and component 2 explained 53.9% and 12.7% of variance, respectively. Obviously, 0% and 20% KCl substitution treatments were far away from others, and 30% and 40% KCl substitution treatments were in the same quadrant because of similar volatile profiles. As shown in Figure 3B, a total of 24 volatile compounds (e.g., 6-methyl-5-hepten-2-one, ethyl caprate, 2-heptanol, nonanal and acetic acid) with VIP > 1 were identified as key volatile compounds [35], which made a great contribution to the difference of volatile profiles among the sausages. Volatile compounds were grouped according to their possible sources, including lipid autoxidation, lipid β-oxidation, esterase activity, carbohydrate metabolism, spices, others and unknown, and the higher proportions of the compounds originating from lipid autoxidation, esterase activity, carbohydrate metabolism and spices were found, accounting for 37.5%, 20.83%, 16.67% and 12.5% respectively.

### 3.7. Sensory Evaluation

**Figure 4:**
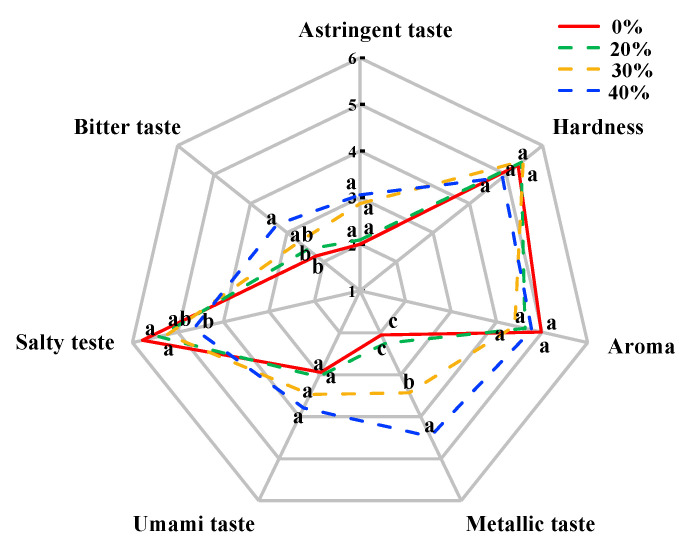
Sensory evaluation of traditional dry sausages with different KCl substitution ratios at day 12. ^a–c^ Means differ significantly among the different treatments (*p* < 0.05). 0%: control dry sausage (2.50% NaCl); 20%: dry sausage with 20% KCl substituted for NaCl (2.00% NaCl + 0.50% KCl); 30%: dry sausage with 30% KCl substitution (1.75% NaCl + 0.75% KCl); 40%: dry sausage with 40% KCl substitution (1.50% NaCl + 1.00% KCl).

The sensory evaluation, including hardness, aroma and taste attributes, of dry sausages after a 12-day fermentation is shown in Figure 4. There were no significant differences in hardness and aroma among treatments (*p* > 0.05), which was consistent with the results of Li et al. [64] and Wu et al. [65]. In terms of taste, there were significant differences among the treatments with high KCl (30% and 40%) and the control treatment (*p* < 0.05) due to the decreased salty taste and the increased bitter taste and metallic taste, which supported the E-tongue results. These undesirable taste characteristics result in negative acceptability from consumer [10]. Notably, no significant difference in umami taste and astringent taste was found among the different treatments (*p* > 0.05), which was different from the result of the E-tongue. This result may be related to sensitivity differences between the human tongue and the E-tongue in these tastes.

### 3.8. Partial Least Squares Regression Analysis

**Figure 5:**
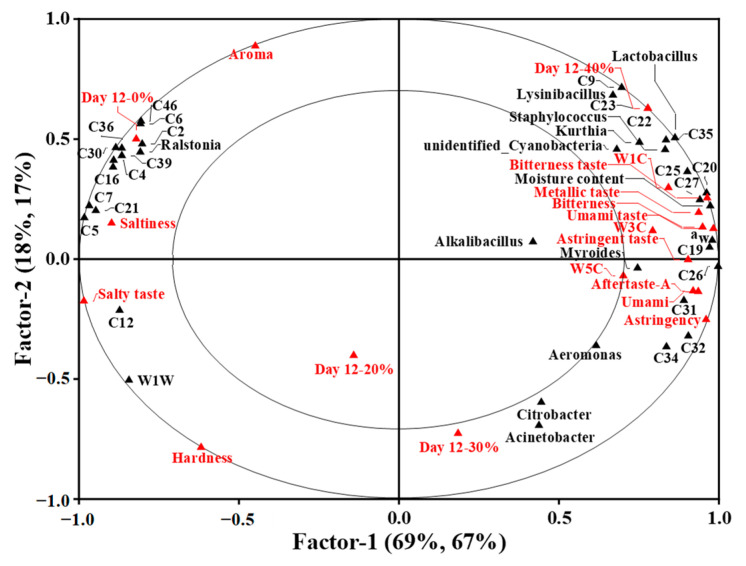
The correlation loading plot for the sausages at day 12 based on partial least squares regression for X-matrix (including physical properties and bacterial counts with significant difference among treatments (*p* < 0.05), dominant bacteria and key volatile compounds) and Y-matrix (including E-nose sensor responses and E-tongue sensor responses with significant difference among treatments (*p* < 0.05), sensory attributes and treatments). 0%: control dry sausage (2.50% NaCl); 20%: dry sausage with 20% KCl substituted for NaCl (2.00% NaCl + 0.50% KCl); 30%: dry sausage with 30% KCl substitution (1.75% NaCl + 0.75% KCl); 40%: dry sausage with 40% KCl substitution (1.50% NaCl + 1.00% KCl).

To investigate the potential correlation between physical and microbial properties, key volatile compounds and sensory attributes of the traditional dry sausages with different KCl substitution ratios at day 12, PLSR analysis was employed. The X-matrix was designed as the physical traits (moisture content and *a*_w_) and bacterial counts with significant differences among treatments, dominant bacteria, and key volatile compounds. The Y-matrix was set as the E-nose sensor responses (W1C, W3C, W5C and W1W sensors) and E-tongue sensor responses (astringency, saltiness, umami, bitterness and aftertaste-A) with significant differences among treatments, sensory attributes and treatments. As shown in Figure 5, the first two factors explained 87% and 84% of X-matrix and Y-matrix variation, respectively, and factor 1 mainly accounted for the variation in X-matrix and Y-matrix. The small ellipse and the big ellipse indicated 50% and 100% of the variance in the correlation loadings plot, respectively. The variables between the two ellipses were well explained by the PLSR model, and the correlation between variables were obtained by their locations.

The dry sausage with 0% KCl located in the left quadrant was correlated to the aroma attribute, which was related to the volatile compounds like humulene (C46), 2-nonanone (C6), nonanal (C2), β-pinene (C39), octadecane (C36), ethyl caprylate (C30), 2-butanone (C4) and terpinen-4-ol (C16), *Ralstonia*, saltiness, salty taste and W1W sensor having high response intensities to 2-heptanol (C12). Dos Santos Alves et al. [40] also observed that sausages without added potassium were described as having a “characteristic aroma and salt taste”. The dry sausages with 20% and 30% KCl located in the lower quadrant were close to each other, indicating that they were similar in overall physical and microbial properties, flavour profiles and sensory attributes. The dry sausages with 40% KCl in the right quadrant were correlated to W1C, W3C and W5C sensors, which had high response intensities to the volatile compounds (e.g., ethyl butyrate (C27), acetic acid (C19), methyl butyrate (C26), heptanoic acid (C20) and ethyl acetate (C25), the bacteria (like *Lysinibacillus*, *Lactobacillus*, *Staphylococcus*, *Kurthia*, unidentified_*Cyanobacteria* and *Myroides*), moisture content, *a*_w_ and the taste defects, such as bitterness taste, metallic taste and astringent taste. This corresponded to sensory evaluation results. Previous studies revealed that sausages with high levels of KCl were characterized by a bitter taste, metallic taste, astringent taste and strange taste [40,66]. Overall, it could be seen that 40% KCl substitution could greatly impairs the quality and taste properties of sausages.

## 4. Conclusions

Increasing the KCl substitution ratio in traditional dry sausages increased the moisture content and relative abundance of *Staphylococcus*, but had little effect on *a*_w_, pH and microbial counts. With the increase in the KCl substitution ratio, the astringency, bitterness and umami gradually increased, and the saltiness gradually decreased. In addition, KCl substitution significantly reduced the concentrations of aldehydes, ketones and some alcohols and increased the concentrations of acids and esters. Sensory evaluation and PLSR indicated that the dry sausages with 30% KCl were similar in overall physical and microbial properties, flavour profiles and sensory attributes, and the sausages with 40% KCl had the characteristic of taste defects. In conclusion, partial substitution of NaCl with 30% KCl for low-sodium dry sausages was favourable for maintaining the characteristic flavour and sensory quality. In future study, we will focus on the effects of the combination of some sodium reduction physical strategies and KCl substitution on the quality characteristics of dry sausages, so as to reduce sodium content to the maximum extent.

## Figures and Tables

**Table 1:** Physical and microbial changes in traditional dry sausage with different KCl substitution ratios during fermentation.

Property	Treatment	Fermentation Time				
		Day 0	Day 3	Day 6	Day 9	Day 12
Moisture content (%)	0%	67.27 ± 1.25 ^Aa^	36.08 ± 0.85 ^Bb^	25.35 ± 0.67 ^Ca^	20.64 ± 0.38 ^Da^	18.02 ± 0.23 ^Db^
	20%	67.72 ± 1.50 ^Aa^	37.10 ± 0.53 ^Bb^	25.15 ± 1.04 ^Ca^	20.44 ± 0.39 ^CDa^	18.67 ± 0.44 ^Dab^
	30%	66.98 ± 1.08 ^Aa^	37.91 ± 0.37 ^Bab^	25.94 ± 0.25 ^Ca^	20.84 ± 1.06 ^Da^	18.84 ± 0.32 ^Dab^
	40%	66.93 ± 1.15 ^Aa^	40.05 ± 0.88 ^Ba^	25.47 ± 0.30 ^Ca^	21.05 ± 0.87 ^Da^	19.95 ± 0.30 ^Da^
*a*_w_	0%	0.98 ± 0.01 ^Aa^	0.88 ± 0.01 ^Ba^	0.77 ± 0.01 ^Ca^	0.67 ± 0.01 ^Da^	0.65 ± 0.01 ^Db^
	20%	0.98 ± 0.01 ^Aa^	0.88 ± 0.01 ^Ba^	0.76 ± 0.01 ^Ca^	0.66 ± 0.01 ^Da^	0.66 ± 0.01 ^Dab^
	30%	0.98 ± 0.01 ^Aa^	0.88 ± 0.01 ^Ba^	0.76 ± 0.01 ^Ca^	0.67 ± 0.01 ^Da^	0.66 ± 0.01 ^Dab^
	40%	0.98 ± 0.01 ^Aa^	0.89 ± 0.01 ^Ba^	0.77 ± 0.01 ^Ca^	0.67 ± 0.01 ^Da^	0.67 ± 0.01 ^Da^
pH	0%	6.18 ± 0.02 ^Aa^	5.78 ± 0.02 ^Ba^	5.72 ± 0.01 ^Ba^	5.74 ± 0.04 ^Ba^	5.78 ± 0.03 ^Ba^
	20%	6.17 ± 0.02 ^Aa^	5.75 ± 0.02 ^Ba^	5.69 ± 0.03 ^BCa^	5.60 ± 0.04 ^Cb^	5.71 ± 0.04 ^BCa^
	30%	6.17 ± 0.01 ^Aa^	5.77 ± 0.01 ^Ba^	5.72 ± 0.01 ^BCa^	5.65 ± 0.01 ^Cab^	5.77 ± 0.04 ^Ba^
	40%	6.17 ± 0.02 ^Aa^	5.78 ± 0.03 ^Ba^	5.66 ± 0.02 ^Ca^	5.68 ± 0.01 ^BCab^	5.69 ± 0.03 ^BCa^
Lactic acid bacteria count (log CFU/g)	0%	3.64 ± 0.03 ^Da^	6.42 ± 0.03 ^Ca^	7.23 ± 0.05 ^Aa^	6.96 ± 0.02 ^ABa^	6.71 ± 0.06 ^BCa^
	20%	3.61 ± 0.04 ^Da^	6.30 ± 0.03 ^Cab^	7.47 ± 0.09 ^Aa^	6.89 ± 0.03 ^Ba^	6.83 ± 0.02 ^Ba^
	30%	3.53 ± 0.03 ^Ea^	6.28 ± 0.02 ^Dab^	7.48 ± 0.05 ^Aa^	7.09 ± 0.06 ^Ba^	6.71 ± 0.10 ^Ca^
	40%	3.60 ± 0.05 ^Da^	6.25 ± 0.03 ^Cb^	7.37 ± 0.12 ^Aa^	6.93 ± 0.08 ^Ba^	6.84 ± 0.03 ^Ba^
Total aerobic count (log CFU/g)	0%	3.86 ± 0.02 ^Da^	6.50 ± 0.05 ^Ca^	7.52 ± 0.02 ^Ab^	7.00 ± 0.05 ^Bb^	6.75 ± 0.10 ^BCa^
	20%	3.95 ± 0.03 ^Da^	6.47 ± 0.05 ^Ca^	7.72 ± 0.07 ^Aa^	7.01 ± 0.05 ^Bb^	6.91 ± 0.11 ^Ba^
	30%	3.78 ± 0.04 ^Ea^	6.39 ± 0.09 ^Da^	7.67 ± 0.02 ^Aab^	7.24 ± 0.03 ^Ba^	6.78 ± 0.08 ^Ca^
	40%	3.80 ± 0.06 ^Da^	6.30 ± 0.08 ^Ca^	7.58 ± 0.03 ^Aab^	7.13 ± 0.04 ^Bab^	7.00 ± 0.05 ^Ba^

^a–b^ Means within the same column with different lowercase letters differ significantly among the different treatments (*p* < 0.05). ^A–E^ Means within the same row with different uppercase letters differ significantly among the different fermentation times (*p* < 0.05). 0%: control dry sausage (2.50% NaCl); 20%: dry sausage with 20% KCl substituted for NaCl (2.00% NaCl + 0.50% KCl); 30%: dry sausage with 30% KCl substitution (1.75% NaCl + 0.75% KCl); 40%: dry sausage with 40% KCl substitution (1.50% NaCl + 1.00% KCl).

**Table 2:** Response values of electronic nose sensors of traditional dry sausage with different KCl substitution ratios at day 0 and day 12.

Sensor Name	Day 0				Day 12			
	0%	20%	30%	40%	0%	20%	30%	40%
W1C	0.11 ± 0.01 ^d^	0.11 ± 0.01 ^d^	0.11 ± 0.01 ^d^	0.11 ± 0.01 ^d^	0.39 ± 0.01 ^c^	0.47 ± 0.01 ^b^	0.42 ± 0.01 ^c^	0.53 ± 0.01 ^a^
W5S	2.14 ± 0.10 ^abc^	2.19 ± 0.07 ^ab^	2.21 ± 0.09 ^a^	2.19 ± 0.07 ^ab^	1.79 ± 0.07 ^cd^	1.83 ± 0.05 ^bcd^	1.79 ± 0.03 ^cd^	1.64 ± 0.02 ^d^
W3C	0.17 ± 0.01 ^c^	0.18 ± 0.01 ^c^	0.17 ± 0.01 ^c^	0.17 ± 0.01 ^c^	0.44 ± 0.01 ^b^	0.51 ± 0.01 ^a^	0.47 ± 0.01 ^b^	0.53 ± 0.01 ^a^
W6S	12.56 ± 0.41 ^a^	12.51 ± 0.40 ^a^	12.78 ± 0.62 ^a^	12.57 ± 0.57 ^a^	3.65 ± 0.14 ^b^	3.25 ± 0.05 ^b^	3.60 ± 0.13 ^b^	2.87 ± 0.14 ^b^
W5C	0.24 ± 0.01 ^c^	0.25 ± 0.01 ^c^	0.24 ±0.01 ^c^	0.24 ± 0.01 ^c^	0.61 ± 0.01 ^b^	0.69 ± 0.01 ^a^	0.64 ± 0.01 ^ab^	0.69 ± 0.01 ^a^
W1S	78.89 ± 4.73 ^a^	77.26 ± 3.40 ^a^	80.07 ± 5.44 ^a^	79.11 ± 5.53 ^a^	20.59 ± 0.51 ^b^	15.13 ± 0.68 ^b^	18.89 ± 0.69 ^b^	14.57 ± 0.50 ^b^
W1W	2.54 ± 0.02 ^a^	2.58 ± 0.01 ^a^	2.59 ± 0.01 ^a^	2.54 ± 0.01 ^a^	1.67 ± 0.01 ^b^	1.64 ± 0.02 ^b^	1.65 ± 0.01 ^b^	1.54 ± 0.01 ^c^
W2S	19.30 ± 0.66 ^a^	18.98 ± 1.10 ^a^	19.55 ± 0.90 ^a^	19.34 ± 0.93 ^a^	4.83 ± 0.12 ^b^	4.09 ± 0.04 ^b^	4.57 ± 0.17 ^b^	3.61 ± 0.07 ^b^
W2W	0.77 ± 0.04 ^a^	0.78 ± 0.01 ^a^	0.76 ± 0.01 ^a^	0.77 ± 0.02 ^a^	0.82 ± 0.01 ^a^	0.82 ± 0.01 ^a^	0.81 ± 0.01 ^a^	0.80 ± 0.01 ^a^
W3S	3.74 ± 0.11 ^a^	3.62 ± 0.15 ^a^	3.83 ± 0.15 ^a^	3.75 ± 0.22 ^a^	1.79 ± 0.02 ^b^	1.64 ± 0.02 ^b^	1.72 ± 0.01 ^b^	1.55 ± 0.02 ^b^

^a–d^ Means within the same row with different lowercase letters differ significantly among the different treatments (*p* < 0.05). 0%: control dry sausage (2.50% NaCl); 20%: dry sausage with 20% KCl substituted for NaCl (2.00% NaCl + 0.50% KCl); 30%: dry sausage with 30% KCl substitution (1.75% NaCl + 0.75% KCl); 40%: dry sausage with 40% KCl substitution (1.50% NaCl + 1.00% KCl).

**Table 3:** Concentration (calculated using the internal standard, µg/kg) of volatile compounds in traditional dry sausage with different KCl substitution ratios at day 0 and day 12.

No.	Volatile Compound	LRI	Day 0	Day 12			
				0%	20%	30%	40%
	Aldehydes (3)						
C1	Hexanal	1084	3.92 ± 0.23 ^d^	3.92 ± 0.23 ^d^	54.52 ± 1.51 ^a^	46.07 ± 1.35 ^b^	48.98 ± 1.24 ^b^
C2	Nonanal	1385	4.91 ± 0.08 ^c^	4.91 ± 0.08 ^c^	18.60 ± 0.60 ^a^	8.72 ± 0.18 ^b^	9.71 ± 0.15 ^b^
C3	Cinnamaldehyde	1847	6.84 ± 0.23 ^b^	6.84 ± 0.23 ^b^	20.97 ± 0.64 ^a^	19.60 ± 0.74 ^a^	18.60 ± 1.03 ^a^
	Ketones (5)						
C4	2-Butanone	974	n.d.	n.d.	16.76 ± 0.77 ^a^	10.20 ± 0.21 ^b^	9.50 ± 0.29 ^b^
C5	3-Hydroxy-2-butanone	1275	2.86 ± 0.21 ^d^	2.86 ± 0.21 ^d^	14.05 ± 0.42 ^a^	11.95 ± 0.32 ^b^	10.64 ± 0.38 ^bc^
C6	2-Nonanone	1336	4.06 ± 0.09 ^c^	4.06 ± 0.09 ^c^	12.75 ± 0.29 ^a^	7.52 ± 0.18 ^b^	6.81 ± 0.17 ^b^
C7	6-Methyl-5-hepten-2-one	1544	11.66 ± 0.32 ^d^	11.66 ± 0.32 ^d^	31.53 ± 1.01 ^a^	27.07 ± 0.74 ^b^	23.52 ± 0.53 ^c^
C8	Fenchone	1083	22.52 ± 0.67 ^c^	22.52 ± 0.67 ^c^	41.72 ± 1.30 ^a^	32.40 ± 1.14 ^b^	26.53 ± 0.92 ^c^
	Alcohols (10)						
C9	Ethanol	928	240.34 ± 4.65 ^a^	240.34 ± 4.65 ^a^	87.08 ± 2.24 ^c^	80.74 ± 1.87 ^c^	83.26 ± 2.68 ^c^
C10	2,3-Butanediol	1590	n.d.	n.d.	15.62 ± 0.33 ^a^	14.00 ± 0.27 ^b^	12.46 ± 0.27 ^c^
C11	2-Ethyl hexanol	1487	10.92 ± 0.54 ^b^	10.92 ± 0.54 ^b^	18.41 ± 0.53 ^a^	12.77 ± 0.31 ^b^	16.87 ± 0.93 ^a^
C12	2-Heptanol	1317	n.d.	n.d.	7.95 ± 0.27 ^a^	4.70 ± 0.15 ^c^	6.37 ± 0.19 ^b^
C13	1-Octen-3-ol	1451	4.28 ± 0.16 ^d^	15.16 ± 0.45 ^a^	11.37 ± 0.30^c^	12.96 ± 0.24^b^	12.14 ± 0.30 ^bc^
C14	Eucalyptol	1033	161.53 ± 3.95 ^b^	314.79 ± 2.48 ^a^	302.52 ± 2.58 ^a^	301.42 ±3.41 ^a^	306.22 ± 4.74 ^a^
C15	Linalool	1552	192.37 ± 2.17 ^c^	251.78 ± 2.38 ^a^	230.55 ± 4.52 ^b^	222.34 ± 5.14 ^b^	239.93 ± 4.02 ^ab^
C16	Terpinen-4-ol	1596	31.26 ± 1.73 ^c^	86.46 ± 2.45 ^a^	64.34 ± 3.03 ^b^	63.49 ± 3.47 ^b^	60.35 ± 2.45 ^b^
C17	α-Terpineol	1688	14.58 ± 0.68 ^c^	23.39 ± 0.80 ^a^	19.32 ± 1.07 ^abc^	16.21 ± 1.18 ^bc^	20.59 ± 1.81 ^ab^
C18	Nerolidol	1546	54.71 ± 3.02 ^b^	75.52 ± 3.83 ^a^	70.77 ± 2.60 ^ab^	70.38 ± 4.48 ^ab^	70.41 ± 4.05 ^ab^
	Acids (5)						
C19	Acetic acid	1450	n.d.	45.45 ± 2.62 ^b^	55.38 ± 1.86 ^ab^	54.64 ± 2.36 ^ab^	63.60 ± 3.34 ^a^
C20	Heptanoic acid	1861	n.d.	13.37 ± 0.35 ^c^	17.23 ± 0.56 ^b^	19.31 ± 0.50 ^b^	28.24 ± 1.12 ^a^
C21	Octanoic acid	2083	n.d.	12.08 ± 0.34 ^a^	9.38 ± 0.50 ^b^	5.51 ± 0.14 ^c^	4.92 ± 0.06 ^c^
C22	2-Ethyl-2-hydroxybutyric acid	1652	n.d.	n.d.	n.d.	n.d.	8.38 ± 0.32
C23	3-Hydroxydecanoic acid	1639	n.d.	n.d.	n.d.	n.d.	9.75 ± 0.41
	Esters (10)						
C24	Methyl acetate	964	n.d.	4.22 ± 0.09 ^c^	4.25 ± 0.12 ^bc^	4.90 ± 0.17 ^a^	4.77 ± 0.08 ^ab^
C25	Ethyl acetate	1002	5.73 ± 0.15 ^d^	9.94 ± 0.41 ^cd^	10.20 ± 0.54 ^c^	25.40 ± 0.78 ^b^	49.77 ± 1.76 ^a^
C26	Methyl butyrate	1065	n.d.	10.53 ± 0.28 ^c^	15.81 ± 0.24 ^b^	17.37 ± 0.49 ^b^	21.38 ± 0.77 ^a^
C27	Ethyl butyrate	1101	n.d.	6.55 ± 0.26 ^c^	15.26 ± 0.47 ^b^	13.52 ± 1.03 ^b^	26.81 ± 1.10 ^a^
C28	Methyl hexanoate	1188	52.97 ± 1.59 ^b^	66.82 ± 1.67 ^a^	69.30 ± 2.83 ^a^	58.85 ± 2.45 ^ab^	67.56 ± 3.64 ^a^
C29	Ethyl hexanoate	1120	44.34 ± 1.87 ^c^	147.47 ± 4.65 ^a^	143.38 ± 3.75 ^ab^	127.88 ± 3.84 ^b^	142.02 ± 5.17 ^ab^
C30	Ethyl caprylate	1437	4.62 ± 0.32 ^d^	17.73 ± 0.87 ^a^	10.38 ± 0.35 ^b^	7.77 ± 0.34 ^c^	8.30 ± 0.29 ^bc^
C31	Ethyl caprate	1634	3.34 ± 0.08 ^c^	12.09 ± 0.52 ^b^	17.11 ± 0.43 ^a^	15.86 ± 0.23 ^a^	18.20 ± 0.92 ^a^
C32	γ-Butyrolactone	1494	n.d.	3.41 ± 0.06 ^b^	9.93 ± 0.29 ^a^	9.72 ± 0.48 ^a^	11.29 ± 0.69 ^a^
C33	Isobornyl acetate	1456	8.12 ± 0.23 ^b^	16.87 ± 0.58 ^a^	16.52 ± 0.39 ^a^	16.44 ± 0.96 ^a^	16.25 ± 0.87 ^a^
	Alkanes (3)						
C34	Pentane	879	n.d.	4.60 ± 0.14 ^c^	6.10 ± 0.21 ^b^	9.81 ± 0.46 ^a^	8.80 ± 0.13 ^a^
C35	Hexane	889	3.03 ± 0.13 ^d^	6.33 ± 0.29 ^c^	5.53 ± 0.28 ^c^	7.95 ± 0.38 ^b^	12.92 ± 0.36 ^a^
C36	Octadecane	1394	3.27 ± 0.40 ^c^	23.78 ± 0.72 ^a^	11.53 ± 0.66 ^b^	11.72 ± 0.46 ^b^	12.88 ± 0.45 ^b^
	Alkenes (14)						
C37	α-Pinene	742	4.87 ± 0.26 ^b^	23.63 ± 0.69 ^a^	23.32 ± 1.17 ^a^	21.80 ± 0.60 ^a^	22.73 ± 1.04 ^a^
C38	Camphene	1705	2.87 ± 0.12 ^c^	22.15 ± 1.33 ^a^	15.09 ± 0.69 ^b^	12.04 ± 0.46 ^b^	15.28 ± 0.41 ^b^
C39	β-Pinene	1116	7.73 ± 0.66 ^c^	23.12 ± 1.21 ^a^	16.69 ± 0.69 ^b^	16.56 ± 0.83 ^b^	16.03 ± 1.15 ^b^
C40	α-Phellandrene	1290	12.20 ± 0.62 ^b^	23.64 ± 1.28 ^a^	22.64 ± 1.82 ^a^	21.52 ± 0.64 ^a^	21.58 ± 0.83 ^a^
C41	β-Myrcene	1186	12.76 ± 0.42 ^c^	52.84 ± 1.44 ^a^	42.15 ± 1.39 ^b^	44.13 ± 1.40 ^b^	43.17 ± 1.14 ^b^
C42	D-Limonene	1209	317.98 ± 8.66 ^c^	885.86 ± 8.56 ^a^	783.83 ± 9.89 ^b^	780.88 ± 8.82 ^b^	779.94 ± 10.68 ^b^
C43	γ-Terpinene	1178	51.29 ± 1.94 ^b^	109.09 ± 5.72 ^a^	107.21 ± 4.08 ^a^	107.70 ± 3.78 ^a^	105.47 ± 3.21 ^a^
C44	Terpinolene	1471	13.73 ± 0.35 ^b^	22.68 ± 0.73 ^a^	21.21 ± 0.64 ^a^	21.19 ± 0.69 ^a^	23.26 ± 0.87 ^a^
C45	Cyclosativene	1400	5.62 ± 0.18 ^b^	12.81 ± 0.63 ^a^	11.22 ± 0.66 ^a^	14.54 ± 0.75 ^a^	13.70 ± 1.34 ^a^
C46	Humulene	1433	3.49 ± 0.10 ^c^	20.66 ± 0.81 ^a^	13.27 ± 0.73 ^b^	11.92 ± 0.34 ^b^	13.12 ± 0.29 ^b^
C47	β-Caryophyllen	1594	127.01 ± 2.91 ^b^	200.17 ± 6.96 ^a^	213.74 ± 2.61 ^a^	209.69 ± 4.71 ^a^	215.07 ± 4.19 ^a^
C48	α-Zingiberene	1532	41.19 ± 1.74 ^b^	65.08 ± 2.51 ^a^	62.37 ± 3.82 ^a^	69.64 ± 3.34 ^a^	71.38 ± 3.86 ^a^
C49	β-Bisabolene	1535	24.44 ± 1.41 ^a^	29.83 ± 1.22 ^a^	30.27 ± 4.93 ^a^	23.38 ± 1.33 ^a^	28.07 ± 1.56 ^a^
C50	α-Curcumene	1773	55.43 ± 1.90 ^a^	63.12 ± 3.84 ^a^	68.26 ± 4.28 ^a^	65.51 ± 2.52 ^a^	69.45 ± 3.50 ^a^
	Others (4)						
C51	Estragole	1507	282.01 ± 4.06 ^b^	356.80 ± 4.59 ^a^	355.89 ± 3.96 ^a^	345.86 ± 4.06 ^a^	344.82 ± 3.77 ^a^
C52	Anethole	1218	535.88 ± 8.25 ^c^	726.47 ± 13.08 ^ab^	732.81 ± 9.79 ^ab^	773.42 ± 12.56 ^a^	685.48 ± 9.24 ^b^
C53	Safrole	1623	25.28 ± 0.72 ^c^	39.84 ± 1.79 ^a^	37.63 ± 1.05 ^ab^	33.73 ± 0.64 ^b^	41.40 ± 1.29 ^a^
C54	Eugenol	2141	105.23 ± 3.76 ^c^	250.52 ± 8.67 ^b^	278.85 ± 6.39 ^a^	240.33 ± 4.91 ^b^	249.48 ± 4.83 ^b^

^a–d^ Means within the same row with different lowercase letters differ significantly among the different treatments (*p* < 0.05). 0%: control dry sausage (2.50% NaCl); 20%: dry sausage with 20% KCl substituted for NaCl (2.00% NaCl + 0.50% KCl); 30%: dry sausage with 30% KCl substitution (1.75% NaCl + 0.75% KCl); 40%: dry sausage with 40% KCl substitution (1.50% NaCl + 1.00% KCl).

## Data Availability

The data presented in this study are available within the article.

## References

[1] Ruusunen M., Puolanne E. (2005). Reducing sodium intake from meat products. Meat Sci..

[2] Tamm A., Bolumar T., Bajovic B., Toepfl S. (2016). Salt (NaCl) reduction in cooked ham by a combined approach of high pressure treatment and the salt replacer KCl. Innov. Food Sci. Emerg..

[3] Quilaqueo M., Duizer L., Aguilera J.M. (2015). The morphology of salt crystals affects the perception of saltiness. Food Res. Int..

[4] World Health Organization (WHO) (2012). Guideline: Sodium Intake for Adults and Children.

[5] He F.J., Tan M., Ma Y., MacGregor G.A. (2020). Salt reduction to prevent hypertension and cardiovascular disease. J. Am. Coll. Cardiol..

[6] World Health Organization (WHO) (2012). Salt Reduction.

[7] Aaslyng M.D., Vestergaard C., Koch A.G. (2014). The effect of salt reduction on sensory quality and microbial growth in hotdog sausages, bacon, ham and salami. Meat Sci..

[8] Campagnol P.C.B., dos Santos B.A., Wagner R., Terra N.N., Pollonio M.A.R. (2011). The effect of yeast extract addition on quality of fermented sausages at low NaCl content. Meat Sci..

[9] de Almeida M.A., Villanueva N.D.M., da Silva Pinto J.S., Saldaña E., Contreras-Castillo C.J. (2016). Sensory and physicochemical characteristics of low sodium salami. Sci. Agric..

[10] Hu Y.Y., Zhang L., Badar I.H., Liu Q., Liu H.T., Chen Q., Kong B.H. (2022). Insights into the flavor perception and enhancement of sodium-reduced fermented foods: A review. Crit. Rev. Food Sci..

[11] Inguglia E.S., Zhang Z.H., Tiwaria B.K., Kerry J.P., Burgess C.M. (2017). Salt reduction strategies in processed meat products-A review. Trends Food Sci. Tech..

[12] Barretto T.L., Bellucci E.R.B., Barbosa R.D., Pollonio M.A.R., Romero J.T., da Silva Barretto A.C. (2020). Impact of ultrasound and potassium chloride on the physicochemical and sensory properties in low sodium restructured cooked ham. Meat Sci..

[13] Castro H., Raij L. (2013). Potassium in hypertension and cardiovascular disease. Semin. Nephrol..

[14] Gelabert J., Gou P., Guerrero L., Arnau J. (2003). Effect of sodium chloride replacement on some characteristics of fermented sausages. Meat Sci..

[15] Gou P., Guerrero L., Gelabert J., Arnau J. (1996). Potassium chloride, potassium lactate and glycine as sodium chloride substitutes in fermented sausages and in dry-cured pork loin. Meat Sci..

[16] Armenteros M., Aristoy M.C., Barat J.M., Toldrá F. (2009). Biochemical changes in dry-cured loins salted with partial replacements of NaCl by KCl. Food Chem..

[17] Gan X., Li H.J., Wang Z.M., Emara A.M., Zhang D., He Z.F. (2019). Does protein oxidation affect proteolysis in low sodium Chinese traditional bacon processing?. Meat Sci..

[18] Wen R.X., Hu Y.Y., Zhang L., Wang Y., Chen Q., Kong B.H. (2019). Effect of NaCl substitutes on lipid and protein oxidation and flavor development of Harbin dry sausage. Meat Sci..

[19] Chen J.X., Hu Y.Y., Wen R.X., Liu Q., Chen Q., Kong B.H. (2019). Effect of NaCl substitutes on the physical, microbial and sensory characteristics of Harbin dry sausage. Meat Sci..

[20] Gan X., Zhao L., Li J.G., Tu J.C., Wang Z.M. (2021). Effects of partial replacement of NaCl with KCl on bacterial communities and physicochemical characteristics of typical Chinese bacon. Food Microbiol..

[21] Yin X.Y., Lv Y.C., Wen R.X., Wang Y., Chen Q., Kong B.H. (2021). Characterization of selected Harbin red sausages on the basis of their flavour profiles using HS-SPME-GC/MS combined with electronic nose and electronic tongue. Meat Sci..

[22] Zhang L., Hu Y.Y., Wang Y., Kong B.H., Chen Q. (2021). Evaluation of the flavour properties of cooked chicken drumsticks as affected by sugar smoking times using an electronic nose, electronic tongue, and HS-SPME/GC-MS. LWT-Food Sci. Technol..

[23] Du H.Z., Chen Q., Liu Q., Wang Y., Kong B.H. (2021). Evaluation of flavor characteristics of bacon smoked with different woodchips by HS-SPME-GC-MS combined with an electronic tongue and electronic nose. Meat Sci..

[24] Hu Y.Y., Zhang L., Zhang H., Wang Y., Chen Q., Kong B.H. (2020). Physicochemical properties and flavour profile of fermented dry sausages with a reduction of sodium chloride. LWT-Food Sci. Technol..

[25] AOAC (1995). Association of Official Methods of Analysis Methods 925.04.

[26] Wen R.X., Lv Y.C., Li X.A., Chen Q., Kong B.H. (2021). High-throughput sequencing approach to reveal the bacterial diversity of traditional yak jerky from the Tibetan regions. Meat Sci..

[27] Brandfass C., Karlovsky P. (2008). Upscaled CTAB-based DNA extraction and realtime PCR assays for *Fusarium culmorum* and *F. graminearum* DNA in plant material with reduced sampling error. Int. J. Mol. Sci..

[28] Chen Q., Hu Y.Y., Wen R.X., Wang Y., Qin L.G., Kong B.H. (2021). Characterisation of the flavour profile of dry fermented sausages with different NaCl substitutes using HS-SPME-GC-MS combined with electronic nose and electronic tongue. Meat Sci..

[29] Zhang D., Li H.J., Emara A.M., Wang Z.F., Chen X.S., He Z.F. (2020). Study on the mechanism of KCl replacement of NaCl on the water retention of salted pork. Food Chem..

[30] Aliño M., Grau R., Toldrá F., Blesa E., Pagána M.J., Barat J.M. (2009). Influence of sodium replacement on physicochemical properties of dry-cured loin. Meat Sci..

[31] Xiao Y.Q., Liu Y.N., Chen C.G., Xie T.T., Li P.J. (2020). Effect of *Lactobacillus plantarum* and *Staphylococcus xylosus* on flavour development and bacterial communities in Chinese dry fermented sausages. Food Res. Int..

[32] Montanari C., Gatto V., Torriani S., Barbieri F., Bargossi E., Lanciotti R., Grazia L., Magnani R., Tabanelli G., Gardini F. (2018). Effects of the diameter on physico-chemical, microbiological and volatile profile in dry fermented sausages produced with two different starter cultures. Food Biosci..

[33] Tangkham W., LeMieux F. (2016). Sensory, physicochemical and microbiological characteristics of venison jerky cured with NaCl and KCl. J. Food Res..

[34] Zhang Q., Chen X.C., Ding Y.T., Ke Z.G., Zhou X.X., Zhang J.Y. (2021). Diversity and succession of the microbial community and its correlation with lipid oxidation in dry-cured black carp (*Mylopharyngodon piceus*) during storage. Food Microbiol..

[35] Lv Y.C., Yin X.Y., Wang Y., Chen Q., Kong B.H. (2021). The prediction of specific spoilage organisms in Harbin red sausage stored at room temperature by multivariate statistical analysis. Food Control..

[36] Shen Y.Y., Wu Y.Y., Wang Y.Q., Li L.H., Li C.S., Zhao Y.Q., Yang S.L. (2021). Contribution of autochthonous microbiota succession to flavor formation during Chinese fermented mandarin fish (*Siniperca chuatsi*). Food Chem..

[37] Perin L.M., Savo S.M.L., Nero L.A., Neviani E., Gatti M. (2017). Bacterial ecology of artisanal Minas cheeses assessed by culture-dependent and -independent methods. Food Microbiol..

[38] Waade C., Stahnke L.H. (1997). Dried sausages fermented with *Staphylococcus xylosus* at different temperatures and with different ingredient levels. Part IV. Amino acid profile. Meat Sci..

[39] Zhao C.J., Schieber A., Gänzle M.G. (2016). Formation of taste-active amino acids, amino acid derivatives and peptides in food fermentations-A review. Food Res. Int..

[40] Dos Santos Alves L.A.A., Lorenzo J.M., Gonçalves C.A.A., dos Santos B.A., Heck R.T., Cichoski A.J., Campagnol P.C.B. (2017). Impact of lysine and liquid smoke as flavor enhancers on the quality of low-fat Bologna-type sausages with 50% replacement of NaCl by KCl. Meat Sci..

[41] Karimi R., Mortazavian A.M., Karami M. (2012). Incorporation of *Lactobacillus casei* in Iranian ultrafiltered Feta cheese made by partial replacement of NaCl with KCl. J. Dairy Sci..

[42] Zhao Y.G., Zhang M., Devahastio S., Liu Y.P. (2019). Progresses on processing methods of umami substances: A review. Trends. Food Sci. Tech..

[43] Medina S., Perestrelo R., Silva P., Pereira J.A.M., Câmara J.S. (2019). Current trends and recent advances on food authenticity technologies and chemometric approaches. Trends. Food Sci. Tech..

[44] Sebzalli Y.M., Wang X.Z. (2001). Knowledge discovery from process operational data using PCA and fuzzy clustering. Eng. Appl. Artif. Intel..

[45] Lorenzo J.M., Carballo J. (2015). Changes in physico-chemical properties and volatile compounds throughout the manufacturing process of dry-cured foal loin. Meat Sci..

[46] Luo J., Nasiru M.M., Zhuang H., Zhou G.H., Zhang J.H. (2021). Effects of partial NaCl substitution with high-temperature ripening on proteolysis and volatile compounds during process of Chinese dry-cured lamb ham. Food Res. Int..

[47] Giri A., Osako K., Okamoto A., Ohshima T. (2010). Olfactometric characterization of aroma active compounds in fermented fish paste in comparison with fish sauce, fermented soy paste and sauce products. Food Res. Int..

[48] Azarbad M.H., Jeleń H. (2015). Determination of hexanal-an indicator of lipid oxidation by static Headspace Gas Chromatography (SHS-GC) in fat-rich food matrices. Food Anal. Method..

[49] Chen Q., Kong B.H., Han Q., Xia X.F., Xu L. (2017). The role of bacterial fermentation in lipolysis and lipid oxidation in Harbin dry sausages and its flavour development. LWT-Food Sci. Technol..

[50] Wu H.Z., Zhuang H., Zhang Y.Y., Tang J., Yu X., Long M., Wang J.M., Zhang J. (2015). Influence of partial replacement of NaCl with KCl on profiles of volatile compounds in dry-cured bacon during processing. Food Chem..

[51] Audrain B., Farag M.A., Ryu C.M., Ghigo J.M. (2015). Role of bacterial volatile compounds in bacterial biology. FEMS Microbiol. Rev..

[52] Sidira M., Kandylis P., Kanellaki M., Kourkoutas Y. (2015). Effect of immobilized *Lactobacillus casei* on the evolution of flavor compounds in probiotic dry-fermented sausages during ripening. Meat Sci..

[53] Ansorena D., Gimeno O., Astiasarán I., Bello J. (2001). Analysis of volatile compounds by GC-MS of a dry fermented sausage: Chorizo de Pamplona. Food Res. Int..

[54] Corral S., Salvador A., Belloch C., Flores M. (2015). Improvement the aroma of reduced fat and salt fermented sausages by *Debaromyces hansenii* inoculation. Food Control..

[55] Stahnke L.H. (1994). Aroma components from dried sausages fermented with *Staphylococcus xylosus*. Meat Sci..

[56] Stahnke L.H. (1995). Dried sausages fermented with *Staphylococcus xylosus* at different temperatures and with different ingredient levels-Part II. Volatile components. Meat Sci..

[57] Lorenzo J.M., Bedia M., Bañón S. (2013). Relationship between flavour deterioration and the volatile compound profile of semi-ripened sausage. Meat Sci..

[58] Talon R., Chastagnac C., Vergnais L., Montel M.C., Berdagué J.L. (1998). Production of esters by *Staphylococci*. Int. J. Food Microbiol..

[59] Wang Y.Q., Li C.S., Zhao Y.Q., Li L.H., Yang X.Q., Wu Y.Y., Chen S.J., Cen J.W., Yang S.L., Yang D.Q. (2020). Novel insight into the formation mechanism of volatile flavor in Chinese fish sauce (Yu-lu) based on molecular sensory and metagenomics analyses. Food Chem..

[60] Zhao J., Wang M., Xie J.C., Zhao M.Y., Hou L., Liang J.J., Wang S., Cheng J. (2017). Volatile flavor constituents in the pork broth of black-pig. Food Chem..

[61] Ahmed E.H., Raghavendra T., Madamwar D. (2010). An alkaline lipase from organic solvent tolerant *Acinetobacter* sp. EH28: Application for ethyl caprylate synthesis. Bioresour. Technol..

[62] Wen R.X., Li X.A., Han G., Chen Q., Kong B.H. (2021). Fungal community succession and volatile compound dynamics in Harbin dry sausage during fermentation. Food Microbiol..

[63] Sharin S.N., Sani M.S.A., Jaafar M.A., Yuswan M.H., Kassim N.K., Manaf Y.N., Wasoh H., Zaki N.N.M., Hashim A.M. (2021). Discrimination of Malaysian stingless bee honey from different entomological origins based on physicochemical properties and volatile compound profiles using chemometrics and machine learning. Food Chem..

[64] Li F., Zhuang H., Qiao W.W., Zhang J.H., Wang Y.L. (2016). Effect of partial substitution of NaCl by KCl on physicochemical properties, biogenic amines and N-nitrosamines during ripening and storage of dry-cured bacon. J. Food Sci. Technol..

[65] Wu H.Z., Zhang Y.Y., Long M., Tang J., Yu X., Wang J.M., Zhang J.H. (2014). Proteolysis and sensory properties of dry-cured bacon as affected by the partial substitution of sodium chloride with potassium chloride. Meat Sci..

[66] Da Silva S.L., Lorenzo J.M., Machado J.M., Manfio M., Cichoski A.J., Fries L.L.M., Morgano M.M., Campagnol P.C.B. (2020). Application of arginine and histidine to improve the technological and sensory properties of low-fat and low-sodium bologna-type sausages produced with high levels of KCl. Meat Sci..

